# Stratifin (14-3-3 σ) Limits Plakophilin-3 Exchange with the Desmosomal Plaque

**DOI:** 10.1371/journal.pone.0077012

**Published:** 2013-10-04

**Authors:** Brett J. Roberts, Roopa Reddy, James K. Wahl

**Affiliations:** Department of Oral Biology, College of Dentistry, University of Nebraska Medical Center, Lincoln, Nebraska, United States of America; University of Colorado, Boulder, United States of America

## Abstract

Desmosomes are prominent cell-cell adhesive junctions in stratified squamous epithelia and disruption of desmosomal adhesion has been shown to have dramatic effects on the function and integrity of these tissues. During normal physiologic processes, such as tissue development and wound healing, intercellular adhesion must be modified locally to allow coordinated cell movements. The mechanisms that control junction integrity and adhesive strength under these conditions are poorly understood. We utilized a proteomics approach to identify plakophilin-3 associated proteins and identified the 14-3-3 family member stratifin. Stratifin interacts specifically with plakophilin-3 and not with other plakophilin isoforms and mutation analysis demonstrated the binding site includes serine 285 in the amino terminal head domain of plakophilin-3. Stratifin interacts with a cytoplasmic pool of plakophilin-3 and is not associated with the desmosome in cultured cells. FRAP analysis revealed that decreased stratifin expression leads to an increase in the exchange rate of cytoplasmic plakophilin-3/GFP with the pool of plakophilin-3/GFP in the desmosome resulting in decreased desmosomal adhesion and increased cell migration. We propose a model by which stratifin plays a role in regulating plakophilin-3 incorporation into the desmosomal plaque by forming a plakophilin-3 stratifin complex in the cytosol and thereby affecting desmosome dynamics in squamous epithelial cells.

## Introduction

Desmosomes are prominent cell-cell adhesive complexes present in stratified squamous epithelial tissues such as skin and the oral mucosa [1,2]. Desmosomal adhesion has important roles in normal tissue architecture and homeostasis. Impairment of this adhesive system, by genetic mutation or by autoimmune mechanisms results in altered tissue morphogenesis and a blistering phenotype respectively [3]. The transmembrane core of the desmosome is comprised of desmosomal cadherins (desmogleins and desmocollins) which interact through their extracellular segments to facilitate cell-cell adhesion. The cytoplasmic domains of the desmosomal cadherins interact with a family of cytoplasmic proteins that assemble to form the desmosomal plaque and recruit the intermediate filament cytoskeleton to sites of cell-cell contact. Plakoglobin, plakin family members such as desmoplakin, and the plakophilins are components of the desmosomal plaque. There are three genes encoding the plakophilins (PKP1-3), and all three plakophilin genes are expressed in the epidermis and oral mucosa [4–6]. Plakophilins are thought to primarily function in two ways. First, plakophilins can cluster desmosomal cadherins in the plane of the plasma membrane [7], and second they are instrumental in recruiting the keratin intermediate filament cytoskeleton to the desmosomal plaque through interactions with desmoplakin [8,9]. While the steady-state composition of the desmosome has been well characterized, the mechanisms that regulate junction assembly and stability are poorly understood.

14-3-3 proteins are a family of seven isoforms that bind phosphoserine and phosphothreonine motifs [10]. This family of proteins can function as homodimers or as heterodimers [11,12]. These proteins participate in a wide range of signal transduction pathways, and multiple 14-3-3 family members often can participate in the same pathway. Stratifin (14-3-3 σ) is a member of the 14-3-3 gene family and plays a role in divergent cell processes including cell cycle regulation, proliferation and differentiation [13–15]. Unlike other 14-3-3 family members, stratifin prefers to homodimerize and associates with signaling partners that are somewhat distinct from that bound by other 14-3-3 family members [10]. Stratifin is primarily expressed in keratinocytes, and it is essential for maintenance of the hair follicle and epidermal homeostasis [15–17]. While stratifin has been shown to bind as many as 130 binding partners [18], a role in regulating cell adhesion has not been established.

In the current study we identified stratifin as a plakophilin-3 interacting partner, and this interaction occurs in the cytosol. As a consequence of binding plakophilin-3, stratifin regulates the exchange of this protein with the desmosomal plaque. Depletion of stratifin results in increased plakophilin-3 exchange with the desmosome, decreased desmosomal adhesive strength and increased cell migration. These results reveal a novel regulatory mechanism affecting desmosome dynamics and may provide new insights into the regulation of cell-cell adhesion.

## Methods

### Cell Culture

The A431 cervical squamous cell carcinoma cell line was purchased from ATCC (Manassas, VA) and cells were grown in DMEM medium (Sigma Chemical Co., St. Louis, MO.) supplemented with 10% fetal bovine serum (Hyclone Laboratories, Logan, UT). UM-SCC-1 cells were obtained from Dr. Thomas Carey (The University of Michigan, Ann Arbor) and were grown in MEM media (Sigma) supplemented with 10% fetal bovine serum [19]. HaCat cells were obtained from Dr. Pam Jensen (University of Pennsylvania, Philadelphia, PA.) and were routinely passaged using DMEM medium lacking calcium (Invitrogen, Grand Island, NY.) supplemented with 10% fetal bovine serum [20]. Prior to extraction or immunofluorescence microscopy cells were grown in DMEM medium containing 1.8 mM calcium for at least 48 hours unless otherwise noted.

### Antibodies and Immunoblotting

Anti plakophilin-3 (11F2) was generated as previously described [21,22] using recombinantly expressed maltose binding protein fused to plakophilin-3 amino acids 1-307 as an antigen. Rabbit anti phospho plakophilin-3 was generated by injecting animals with the peptide APSVRSL[pS]LSLADS conjugated to KLH (21^st^ Century Biochemicals, Marlboro, MA.). Specific IgG recognizing the phosphorylated peptide was affinity purified and IgG recognizing the unphosphorylated peptide was removed. Anti-green fluorescent protein (GFP) was purchased (Clontech, Mountain View, CA.). Anti c-myc (9E10), anti-beta-tubulin (E7) and anti stratifin (CPTC-SFN-1) were purchased from The Developmental Studies Hybridoma Bank developed under the auspices of the NICHD and maintained by The University of Iowa, Department of Biology, Iowa City, IA 52242. Immunoprecipitations, preparation of cell lysates and immunoblotting was performed as previously described [23–26].

*Molecular constructs*. cDNA encoding human plakophilin-3 (Genbank accession number BC000081) and stratifin (Genbank accession number BC000995) were purchased from Open Biosystems (Huntsville, AL). PCR was used to add sequence encoding a 2x c-myc epitope tag to the 5’ end of the open reading frame of plakophilin-3 to generate Pkp-3-myc. To generate Pkp-3/HD-myc, additionally a stop codon was introduced following the codon encoding amino acid 307. Pkp-3/arm-myc was generated by PCR adding a myc tag immediately 5’ to sequences coding for amino acids 308-797. Generation of plakophilin-3/GFP has been previously described [23]. All PCR products were sequenced to verify that no unintended mutations were introduced by Taq polymerase.

Shorthairpin RNAs (shRNA) targeting stratifin mRNA were generated by synthesis of oligonucleotides per the pSuper retro neo user manual (Oligoengine, Seattle, WA.) using the gene specific sequence 5’- ACCTGAAGATGAAGGGTGA-3’. Control shRNA was prepared using the sequence 5’-GAACATGCTGGGAACCTTATT-3’. Oligos were annealed and ligated to pSuper retro neo digested with BglII and HindIII. The shRNA construct was sequenced to verify that the correct oligonucleotides were synthesized and ligated to pSuper retro-neo. Retroviral particles were generated and stable cell lines were selected as previously described [9].

### Detergent extraction and immunoprecipitation

Cell lysates for immunoprecipitation and western blot analysis were prepared as previously described [9,26]. For the generation of soluble, membrane and SDS soluble fractions, cell monolayers were washed three times using phosphate buffered saline and cells were scraped from the dish in buffer without detergent (10 mM Tris HCl, pH 7.5, 150 mM NaCl, and 2 mM EDTA) with protease inhibitor cocktail (Sigma Chemical Co.). The membrane fraction was collected by centrifugation (14,000 x g for 10 minutes) and extracted in buffer containing 0.5% Triton X-100. The Triton X-100 insoluble material was collected by centrifugation (14,000 x g for 10 minutes) and was re-suspended in buffer containing 2% SDS. In order to immunoprecipitate plakophilin-3 from SDS containing lysates, the lysate was diluted to 0.1% SDS using buffer containing no detergent prior to adding the lysate to antibody coated beads. Immunoprecipitation reactions were assembled by absorbing mouse anti plakophilin-3 or anti stratifin IgG on anti-mouse conjugated affinity gel (MP Biomedical, Santa Ana, CA). Control immunoprecipitation reactions omitted the immunoprecipitating antibody from the reaction. Unbound antibody was removed by washing the beads with lysis buffer and beads were incubated with lysate containing approximately 1 mg of total cellular protein. Immune complexes were washed five times using TBST (10 mM Tris HCl, pH 8.0, 150 mM NaCl, 0.05% Tween-20) and the final pellet was re-suspended in 2 x Laemmli sample buffer.

### Yeast Two-hybrid assays

Protein-protein interactions were tested using the Matchmaker 3 system from Clontech (Palo Alto, CA). Plakophilin bait constructs were subcloned into pGBKT7 to create Gal4 DNA binding domain fusion proteins. PCR was used to generate the bait plasmids Pkp-1 amino acids 1-234, Pkp-2 amino acids 1-342, Pkp-3 amino acids 1-307, Pkp-3 amino acids 308-797. Stratifin cDNA was subcloned into pGADT7 to create a prey plasmid encoding full-length Stratifin fused to the Gal4 activation domain (AD). Desmoplakin (DP) head domain fused to Gal4 AD was previously described [9]. All PCR generated cDNAs were sequenced to verify no mutations were introduced by Taq polymerase. Two-hybrid assays were performed according to the manufacturer’s protocol.

### Dispase assays

Cells were grown to confluence in 6-well dishes and 24 hours post confluence the cell sheets were removed from the culture dish by incubating with dispase (Roche Applied Science, Indianapolis, IN.). Cell sheets were carefully transferred to 15 mL conical tubes and subjected to mechanical stress by inversion. Cell sheet fragments were counted, assays were performed in triplicate and the data are expressed as the average number of fragments/well.

### Cell motility assays

Cell migration assays were performed by plating 2.8 x 10^4^ cells in 24 well dishes containing Ibidi Culture-Inserts (Verona, WI.). Cells were cultured 48 hours before removing the insert and imaging cell migration. Boyden chamber in vitro motility assays were performed as described by Nieman et al. [27]. 5 x 10^5^ cells were plated in the top chamber of non-coated polyethylene tetraphthalate (PET) membranes (6-well insert, pore size 8 mm; BD Biosciences). Cells were incubated for 24 hours and cells that did not migrate through the membrane were scraped from the membrane using a cotton swab. Cells that migrated to the bottom of the membrane were fixed and stained to facilitate counting. Cells in ten random fields of view were counted and the data are expressed as the average number of cells/field of view. Three independent experiments were done in each case. The data are represented as the average of the three independent experiments and the standard deviation of the average is indicated.

### Immunofluorescence Microscopy and FRAP analysis

Cells grown on glass cover slips were washed briefly in Hepes buffered Hanks’ balanced salts solution (HHBSS) and fixed in 1% formaldehyde prepared from paraformaldehyde in HHBSS. Antibody staining was performed as previously described in [9,28]. Fluorescence recovery after photobleaching (FRAP) was performed using cells expressing plakophilin-3/GFP and plakophilin-3 ΔSL/GFP fusion proteins. GFP fluorescence at cell-cell borders was photobleached and recovery was measured using a Marianas Live Cell microscopy system (Intelligent Imaging Innovations Inc, Denver, Co.) equipped with a Stanford Research Systems laser ablation system (model NL100). After photobleaching, z-stack images were collected every 10 seconds for 15-20 minutes. Raw images were deconvolved and collapsed into a projection image prior to determination of relative fluorescence intensity. In order to compare multiple FRAP experiments (n=13 for each cell line), normalized FRAP data was collected using SlideBook 5.0 and relative fluorescence intensity values were determined by setting the pre-bleach value as 100% and the post-bleach value was set to 0. For each graph, the relative fluorescence intensity is plotted and the error bars represent the 95% confidence interval.

## Results

**Figure 1:**
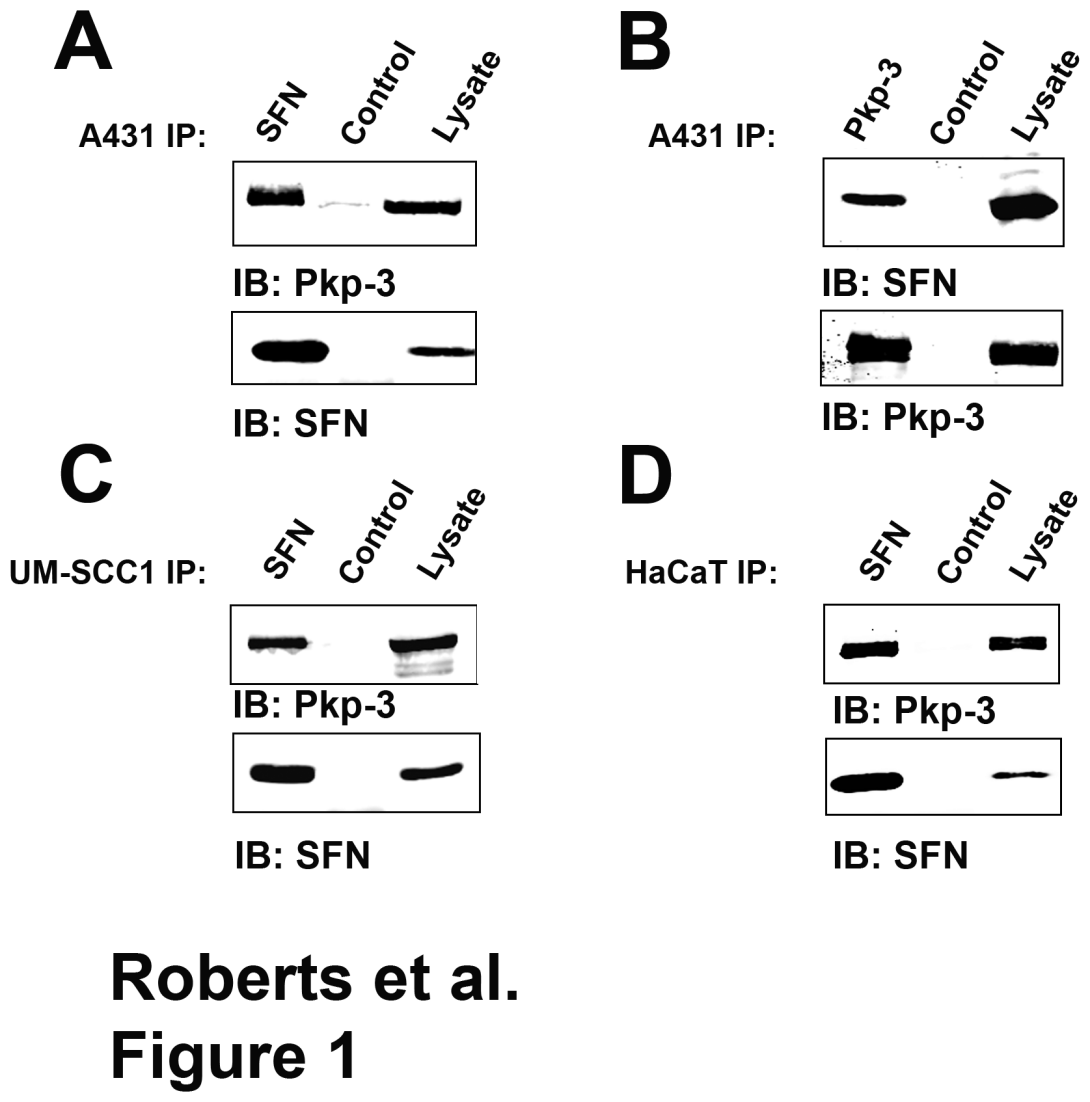
Plakophilin-3 and stratifin form a complex in cultured cells. A. Stratifin was immunoprecipitated from lysate prepared from A431 cells, the immunoprecipitate was resolved by SDS-PAGE and immunoblotted (IB) using an anti plakophilin-3 antibody (top) and anti stratifin (bottom). Lanes marked “Control” are immunoprecipitation reactions lacking the primary antibody. B. The reverse immunoprecipitation was performed using anti plakophilin-3 to immunoprecipitate the complex. The immunoprecipitate was resolved by SDS-PAGE and immunoblotted using anti stratifin (top) and anti plakophilin-3 (bottom). Additionally, stratifin was immunoprecipitated from cell lysates prepared from (C) UM-SCC-1 and (D) HaCat cells and the immunoprecipitates immunoblotted using anti plakophilin-3 (top) and anti stratifin (bottom).

In an effort to identify novel plakophilin-3 interacting proteins, we immunopurified myc-tagged plakophilin-3 from A431 cell lysates and identified potential interacting partners by mass spectroscopy. Plakophilin-3 immune complexes were trypsin digested and peptides were identified by mass spectroscopy. One of the interacting proteins identified was stratifin (14-3-3 σ). We verified our mass spectroscopy result by co-immunoprecipitating endogenously expressed plakophilin-3 and stratifin from parental A431 cell lysate prepared from confluent cells (Figure 1). We found that stratifin co-immunoprecipitated with endogenously expressed plakophilin-3 in A431 cells and reciprocal immunoprecipitation demonstrated that plakophilin-3 co-immunoprecipitated with stratifin. We were also able to co-immunoprecipitate the plakophilin-3/stratifin complex from UM-SCC-1 and HaCat cells demonstrating this interaction is not restricted to A431 cells. These data are in agreement with a previous study in which plakophilin-3 was identified as a stratifin interacting partner in a large scale proteomics study examining stratifin interacting partners [29]. While stratifin has been shown to interact with an enormous number of cellular proteins, this is the first characterization of the interaction of stratifin with the desmosomal plaque component, plakophilin-3.

**Figure 2:**
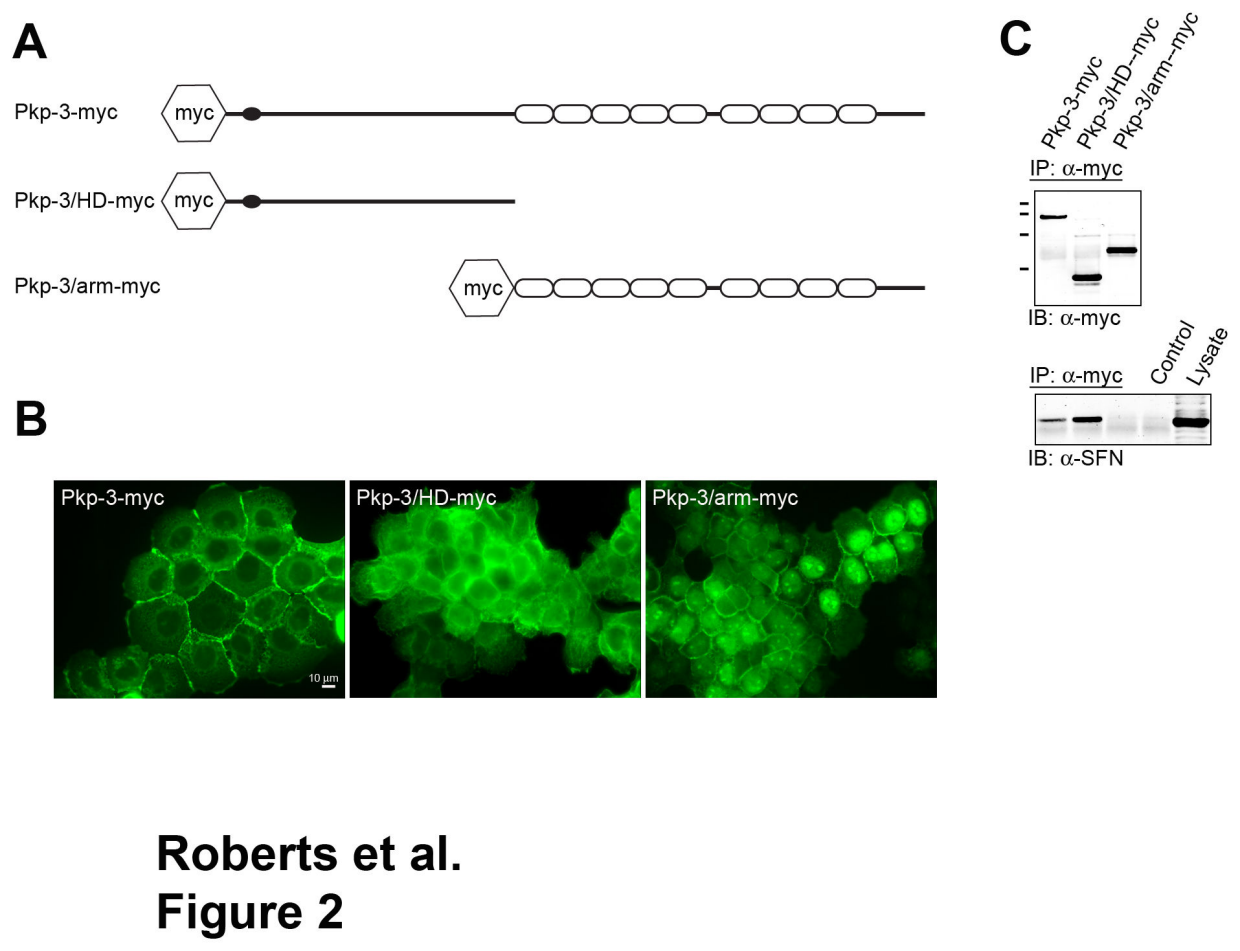
Stratifin interacts with the amino terminal domain of plakophilin-3. A. Retroviral expression vectors were generated encoding epitope-tagged plakophilin-3 (Pkp-3-myc), plakophilin-3 amino acids 1-307 (Pkp-3/HD-myc) and plakophilin-3 amino acids 308-797 (Pkp-3/arm-myc). B. Immunofluorescence microscopy using an anti c-myc antibody (9E10) revealed the subcellular localization of the exogenous plakophilin-3 proteins stably expressed in A431 cells. C. (upper panel) Immunoblot analysis of plakophilin-3 immunoprecipitated from cell lysates prepared from A431 cells expressing the plakophilin-3 demonstrate the exogenous proteins are immunoprecipitated at approximately equal levels, and the proteins migrate at the appropriate molecular mass by SDS-PAGE (MW markers from top to bottom; 116 kDa, 97 kDa, 68 kDa, and 45 kDa). (lower panel) The immunoprecipitates were immunoblotted using anti stratifin. Note that full length Pkp-3-myc and the Pkp-3/HD-myc co-immunoprecipitated stratifin while Pkp-3/arm-myc did not.

In order to map the domain on plakophilin-3 that interacts with stratifin, we expressed myc-tagged fragments of plakophilin-3 in A431 cells and examined the ability of these fragments to co-immunoprecipitate endogenous stratifin. Epitope-tagged plakophilin-3, plakophilin-3 head domain (HD) and plakophilin-3 armadillo repeat domain (arm) proteins were expressed in A431 cells, stable G418 resistant populations were generated and plakophilin-3 localization was determined by immunofluorescence microscopy using an anti c-myc antibody (Figure 2A).

Full-length plakophilin-3 (Pkp-3-myc) localized to sites of cell-cell contact in a punctate staining pattern indicative of desmosome localization (Figure 2B). In addition, a significant cytoplasmic pool of plakophilin-3 was detected while very little nuclear localization was observed. The plakophilin-3 head domain (Pkp-3/HD-myc) protein was not found to co-localize with endogenous plakophilin-3 at sites of cell-cell contact and was entirely cytoplasmic. The plakophilin-3 armadillo repeat domain (Pkp-3/arm-myc) localized to cell-cell borders as well as to the nucleus, however the nuclear localization was not homogeneous throughout the culture. The localization of plakophilin-3 fragments was similar to that of transiently expressed fragments of plakophilin-3 reported previously, however cytoplasmic aggregates [30] were not observed in the cells stably expressing the plakophilin-3 proteins.

Myc tagged plakophilin was immunoprecipitated from A431 cell lysates and immunoblot analysis using anti c-myc shows the amount of plakophilin-3 immunoprecipitated is approximately equal in each immunoprecipitation (Figure 2C upper panel). Stratifin was found to co-immunoprecipitate with full-length plakophilin-3 and the plakophilin-3 head domain but not with the armadillo repeat domain (Figure 2C lower panel).

**Figure 3:**
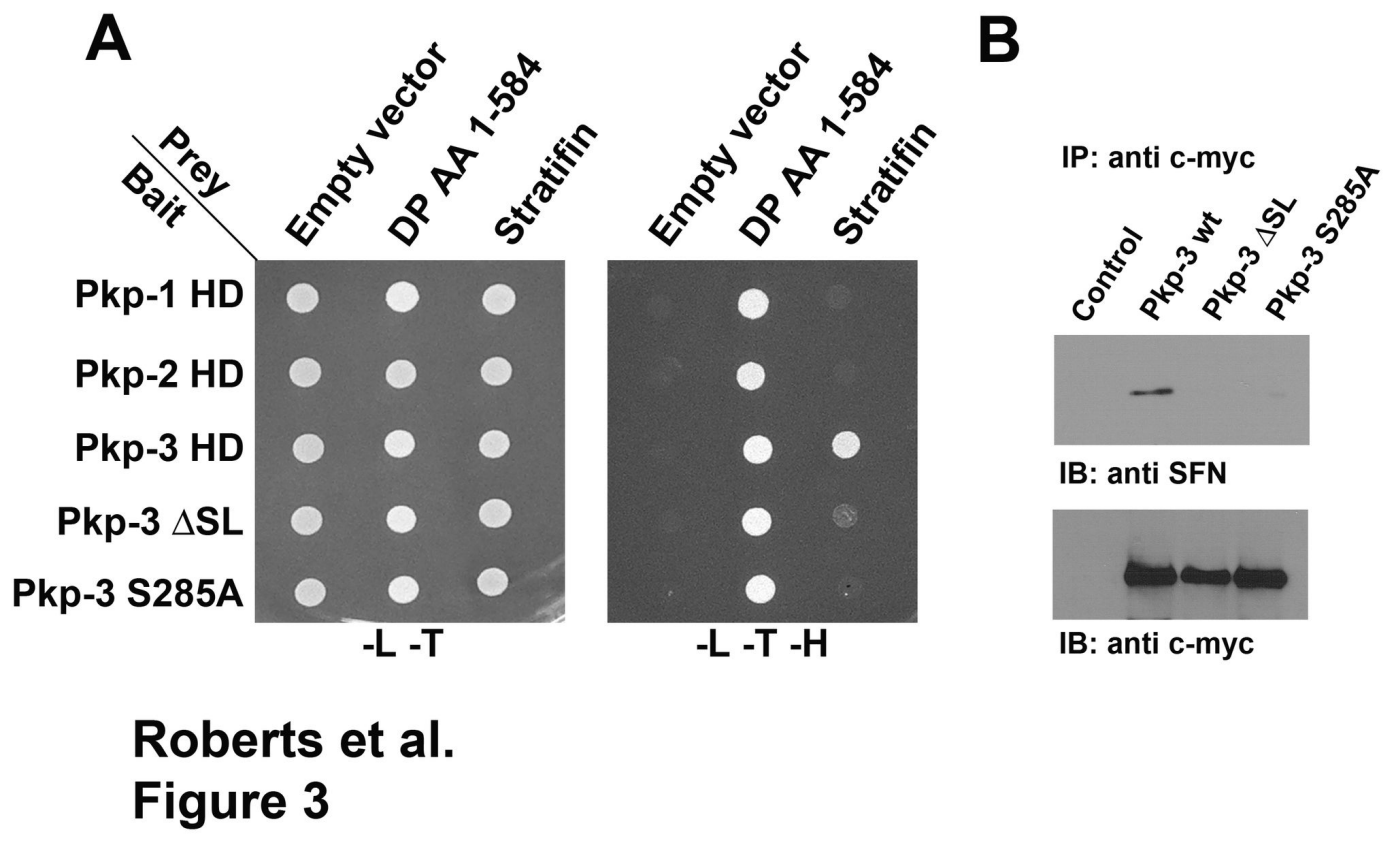
Stratifin interacts directly with plakophilin-3 but not with plakophilin-1 or plakophilin-2. A. Y2H Gold yeast cells were doubly transformed with “bait” and “prey” plasmids. The bait plasmids (pGBKT7) were plakophilin-1 HD (amino acids 1-234), plakophilin-2 HD (amino acids 1-342), plakophilin-3 HD (amino acids 1-307), plakophilin-3 HD ΔSL lacking amino acids 283-287) or plakophilin-3 HD S285A. The prey plasmids were empty vector (pGADT7), desmoplakin (amino acids 1-584) or full-length stratifin. All the transformants grew on selection plates lacking leucine and tryptophan indicating they contained both plasmids. Activation of the histidine reporter was assessed by cell growth on plates lacking histidine. Note that desmoplakin interacted with all the plakophilin bait proteins while only wild-type plakophilin-3 HD interacted with stratifin. B. Cell lysates were immunoprecipitated using anti-myc from A431 cells (control) and A431 cells expressing myc-tagged full-length plakophilin-3, plakophilin-3 ΔSL or plakophilin-3 S285A. Immunoblot analysis using anti stratifin demonstrated that stratifin co-immunoprecipitated with wild-type plakophilin-3 but not plakophilin-3 ΔSL or S285A.

Yeast two-hybrid analysis was used to confirm that the interaction of plakophilin-3 and stratifin was direct. Bait proteins encoding plakophilin head domains (Pkp-1, 2, and 3) fused to the Gal4 DNA binding domain were constructed in pGBKT7. A prey plasmid encoding full-length human stratifin fused to the Gal4 activation domain was assembled in pGADT7. The amino terminal domain of human desmoplakin was previously shown to interact with the plakophilin head domains and was used as a positive control in this experiment. The bait and prey plasmids were co-transformed and the ability of co-transformants to grow on media lacking leucine, tryptophan and histidine was assessed. Yeast colonies co-expressing either plakophlin-1 or plakophilin-2 construct together with the stratifin prey construct failed to grow on selective media. Yeast co-transformed with the plakophilin-3 head domain and stratifin were able to grow on selective media indicating the two proteins directly interact (Figure 3A). The plakophilin-3 armadillo repeat domain failed to interact with stratifin consistent with the co-immunoprecipitation experiment above.

Examination of plakophilin-3 amino acids 1-307 using Scansite Motif Scan [31] revealed a potential 14-3-3 interaction motif at amino acids 283-288 (SL**S**LS). Interaction of 14-3-3 proteins with binding partners is often dependent on phosphorylation of specific serine residues within the binding motif. The prediction algorithm predicted serine 285 as a potential phosphoserine residue capable of mediating the interaction with 14-3-3 proteins. Deletion of amino acids 283-288 (ΔSL) and mutation of serine 285 to alanine (S285A) abrogated the interaction of plakophilin-3 with stratifin while these plakophilin-3 mutants were still capable of interacting with desmoplakin (Figure 3A).

We confirmed our yeast two-hybrid results by expressing full-length myc tagged plakophilin-3 mutants lacking amino acids 283-288 (ΔSL) or a mutant plakophilin-3 harboring the serine 285 to alanine mutation. In addition, wild-type plakophilin-3 co-immunoprecipitated stratifin while the mutant plakophilin-3 proteins failed to co-immunoprecipitate stratifin from cell lysates expressing these mutants. Taken together these data demonstrate that plakophilin-3 specifically interacts with stratifin and this interaction is dependent on serine 285 (Figure 3B).

### Stratifin interacts with the cytoplasmic pool of plakophilin-3

**Figure 4:**
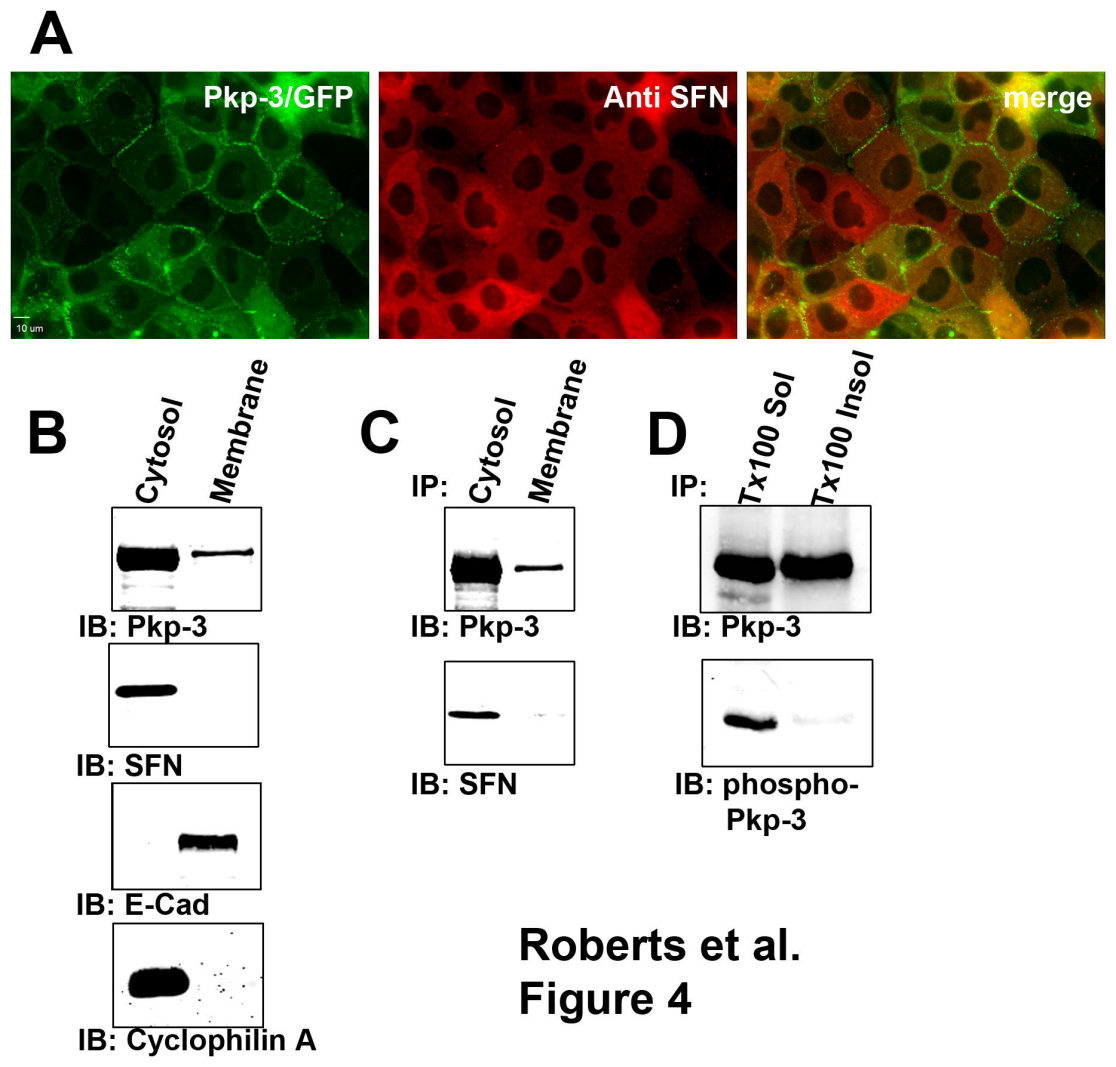
Stratifin interacts with plakophilin-3 in the cytosol. A. A431 expressing plakophilin-3/GFP were fixed and stained with antibodies against stratifin. Note the localization of stratifin in the cytoplasm and a lack of co-localization of plakophilin-3/GFP and stratifin at cell-cell borders. B. parental A431 cells were lysed and the lysates separated into cytosolic (dounce homogenized cytosolic fraction) and membrane extracts (Triton X-100 soluble fraction). Immunoblot analysis revealed plakophilin-3 was present in the cytosolic fraction as well as the Triton X-100 soluble membrane fraction. Immunoblot analysis revealed stratifin was only detectable in the cytosolic fraction. Cytosol fraction and membrane fractions were verified by immunoblot analysis with anti E-cadherin and anti cyclophilin A. C. Endogenous plakophilin-3 was immunoprecipitated from A431 cytosolic fraction and A431Triton X-100 soluble membrane fraction. Immunoblot analysis revealed stratifin co-immunoprecipitated with plakophilin-3 from the cytoplasmic fraction but not from the membrane fraction. D. Plakophilin-3 was immunoprecipitated from Triton X-100 soluble fraction and from SDS extracted membrane fraction (Triton X-100 insoluble fraction). Immunoprecipitates were immunoblotted using anti plakopihilin-3 and anti phospho plakophilin-3 (pSer285).

Plakophilin-3 is normally localized to the desmosomal plaque as well as the cytoplasm [4,30]. Additionally, overexpressed fragments of plakophilin-3 can localize to the nucleus in many cultured epithelial cells [30]. Stratifin localization was examined in A431 cells expressing plakophilin-3/GFP. Stratifin localized exclusively to the cytoplasm and is not detected in desmosomal junctions at the plasma membrane or in the nucleus (Figure 4A). Additionally, stratifin was not found to co-localize with any distinguishable cytosolic structures in A431 cells. Plakophilin-3/GFP was localized to the cytosol and the desmosome in A431 cells in a manner indistinguishable from that of endogenous plakophilin-3 [23]. Plakophilin-3/GFP appears to partially overlap with that of endogenous stratifin in the cytosol. Subcellular fractionation and immunoblot analysis of parental A431 cells revealed that plakophilin-3 is found in a cytosolic pool as well as in a membrane-bound pool while stratifin is exclusively present in the cytoplasmic pool (Figure 4B). Plakophilin-3 was immunoprecipitated from detergent-free dounce homogenates and Triton X-100 soluble membrane fractions (Figure 4C). Stratifin was detected in immunoprecipitates from the cytoplasmic dounce homogenate but was not detected in the immunoprecipitate from the membrane fraction. Additionally, we expect that phosphorylated plakophilin-3 would be present in the cytosol and not in the membrane fraction. Immunoprecipitation of equal amounts of plakophilin-3 from the Triton X-100 soluble fraction and the SDS solubilized membrane fraction revealed phosphorylated plakophilin-3 was present in the Triton X-100 soluble fraction but was not detected in the membrane fraction.

### Stratifin expression increases plakophilin-3 exchange with the desmosomal plaque

**Figure 5:**
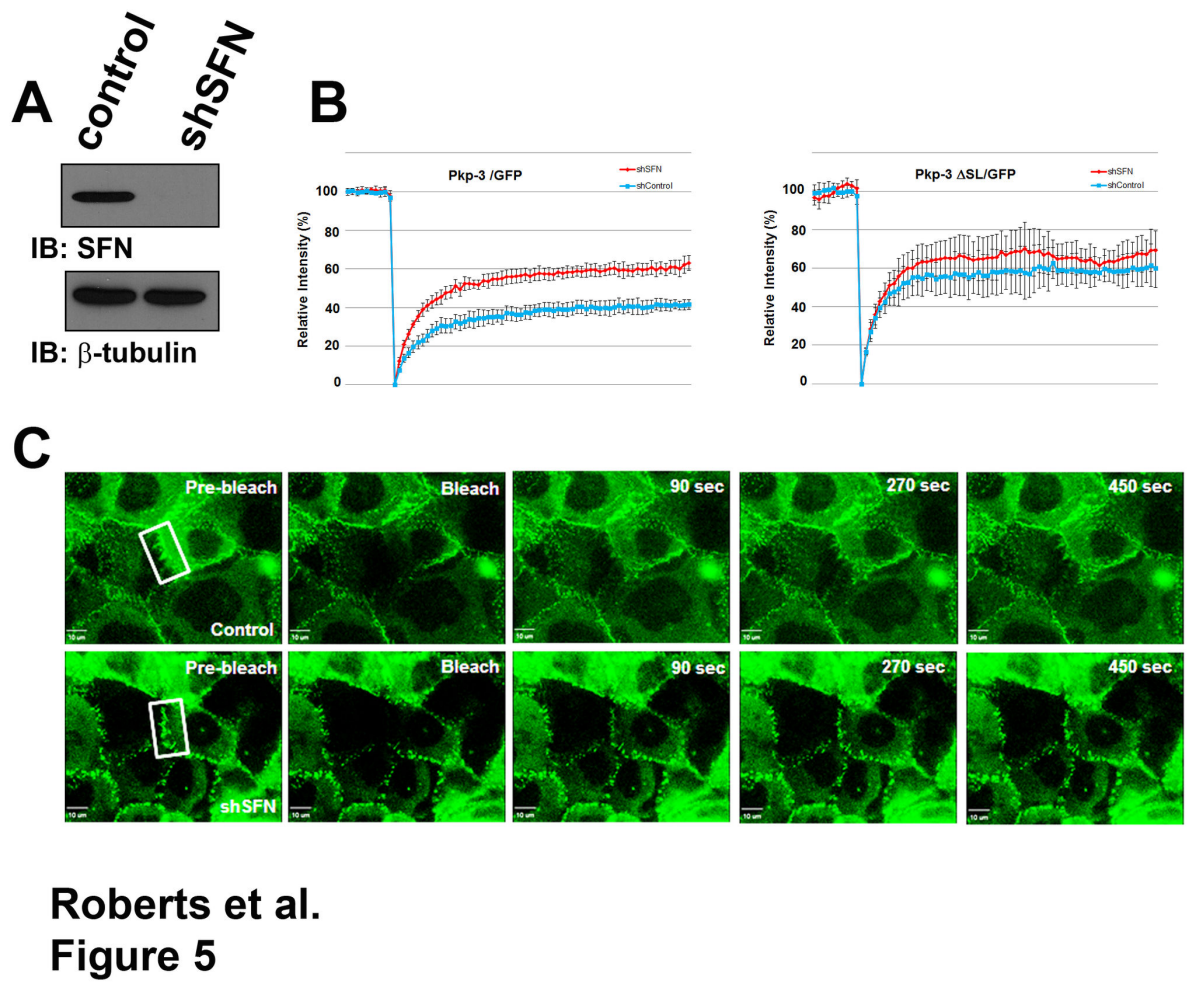
Decreased stratifin expression results in increased exchange of plakophilin-3/GFP at the plasma membrane. A. Cell lysate prepared from A431 cells expressing a control shRNA and A431 cells stably expressing shSFN were immunoblotted with anti stratifin and anti beta-tubulin. B. FRAP analysis of A431 cells expressing plakophilin-3/GFP together with a control shRNA or shRNA targeting stratifin (left panel) and A431 cells expressing plakophilin-3 ΔSL/GFP together with control shRNA or shRNA targeting stratifin (right panel). For each graph, the relative fluorescence intensity is plotted and the error bars represent 95% confidence interval. C. Representative images (Movies S1 and S2) from the FRAP experiment depicted in the left panel of B.

Neither overexpression nor knock down of stratifin in A431 and HaCat cells affected steady state desmosome assembly visualized by standard immunofluorescence microscopy. Additionally, plakophilin-3 protein stability and plakophilin-3 localization at the plasma membrane was unaffected by decreased stratifin expression (data not shown). Furthermore, expression of plakophilin-3 had no effect on stratifin expression and stability. Since stratifin and plakophilin-3 form a complex in the cytosol, we examined the possibility that stratifin regulates the dynamic incorporation of plakophilin-3 into the desmosomal plaque. We propose that stratifin interacts with the cytoplasmic pool of plakophilin-3 and limits the availability of this pool to exchange with the desmosome. Decreased expression of stratifin should increase the pool of plakophilin-3 available to exchange with the desmosomal plaque. To test this hypothesis we expressed plakophilin-3/GFP [23] in A431 cells and examined plakophilin-3 exchange at the desmosomal plaque by FRAP analysis. Following photobleaching at the cell border, plakophilin-3/GFP in the cytosol exchanged with the bleached plakophilin-3/GFP in the desmosomal plaque and 42% (+/- 4.7%) of the fluorescence intensity recovered at 20 minutes after bleaching (Figure 5B). This analysis reveals that a fraction of plakophilin-3/GFP is dynamically exchanging with the desmosomal plaque while another pool of plakophilin-3 is stably incorporated into the desmosome and is unable to exchange with the cytoplasmic pool of plakophilin-3.

We generated shRNA constructs targeting stratifin and expressed these shRNAs in A431 cells expressing plakophilin-3/GFP (Figure 5A). A431 cells expressing shRNAs targeting stratifin exhibited increased exchange of plakophilin-3/GFP with the desmosomal plaque. In the absence of stratifin, plakophilin-3/GFP recovered to approximately 60% (+/- 6.0%). In order to confirm the increase in exchange with the desmosomal plaque was due to interactions with stratifin, we examined the exchange of plakophilin-3 ΔSL/GFP that lacks the stratifin binding site. This plakophilin-3 mutant exhibited increased exchange with the desmosomal plaque compared to wild type plakophilin-3 and decreased expression of stratifin did not significantly alter the exchange of plakophilin-3 ΔSL/GFP with the desmosomal plaque demonstrating that plakophilin-3 ΔSL/GFP is insensitive to stratifin regulation. While these FRAP assays allow us to examine the exchange of plakophilin-3/GFP fusion proteins, we expect that decreased stratifin expression has similar effects on the endogenous plakopiln-3 expressed by A431 cells. These data suggest a model whereby stratifin interacts with plakophilin-3 in the cytoplasm and regulates the dynamic exchange of cytoplasmic plakophilin-3 and desmosomal plakophilin-3.

### A431 cells expressing plakophiln-3 mutants display decreased desmosomal adhesion and increased motility

**Figure 6:**
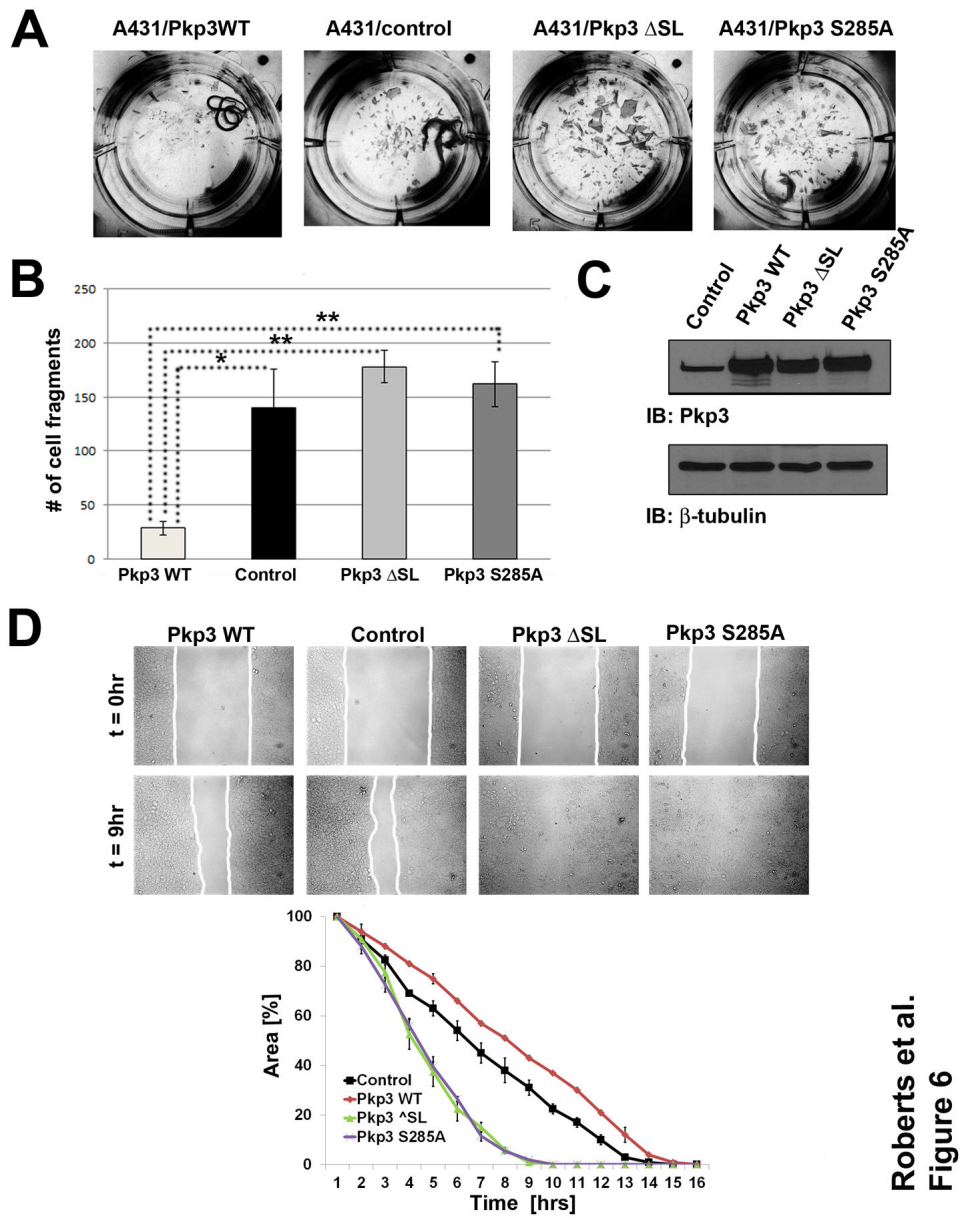
Cells expressing of plakophilin-3 mutants display decreased desmosomal adhesion and increased migration compared to cells expressing wild type plakophilin-3. Desmosomal adhesion was measured using the dispase adhesion assay. Cells expressing wild type plakophilin-3 and plakophilin-3 mutants (ΔSL and S285A) were compared to control A431 cells (panels A and B). Expression of myc tagged plakophilin-3 and plakophilin-3 mutants in A431 cells were verified by immunoblot analysis of cell lysates from each stable cell line. Immunoblot of the lysates with anti-beta tubulin is shown as a loading control (panel C). Cell migration was measured by observing the time required to fill a cell free gap generated by a silicone insert. The identical field of view was imaged every hour for 16 hours and the percent area unfilled was calculated (panel D).

We generated A431 cells stably expressing plakophilin-3 mutants (S285A and DSL) and examined desmosomal adhesion and cell migration of these cell lines compared to A431 cells expressing wild type plakophilin-3. Desmosomal adhesion was measured using a dispase adhesion assay [32,33]. Expression of wild type plakophilin-3 in A431 cells results in an increase in desmosomal adhesion and a decrease in the fragmentation of cell sheets after mild mechanical shearing compared to control A431 cells (Figure 6A and B). A431 cells expressing plakophilin-3 mutants did not display an increase in desmosomal adhesion and were not significantly different than control A431 cells (Figure 6B).

Additionally, we examined the migration of A431 cells expressing the plakophilin-3 mutants and compared these cell lines to A431 cells expressing wild type plakophilin-3 and control A431 cells. Cells were plated in the presence of a small silicone insert and the insert was removed using sterile forceps after the cells reached confluence. The migration of the cells into the clear area of the dish was monitored and photographed. A431 cells expressing plakophilin-3 mutants migrated more quickly than control A431 cells and A431 cells expressing wild type plakophilin-3 (Figure 6D). Taken together these data demonstrate that cells expressing plakophilin-3 mutants unable to associate with stratifin display decreased desmosomal adhesion and increased cell migration compared to cells expressing wild type plakophilin-3.

### Decreased stratifin expression decreased desmosomal cell-cell adhesion and increased cell migration

**Figure 7:**
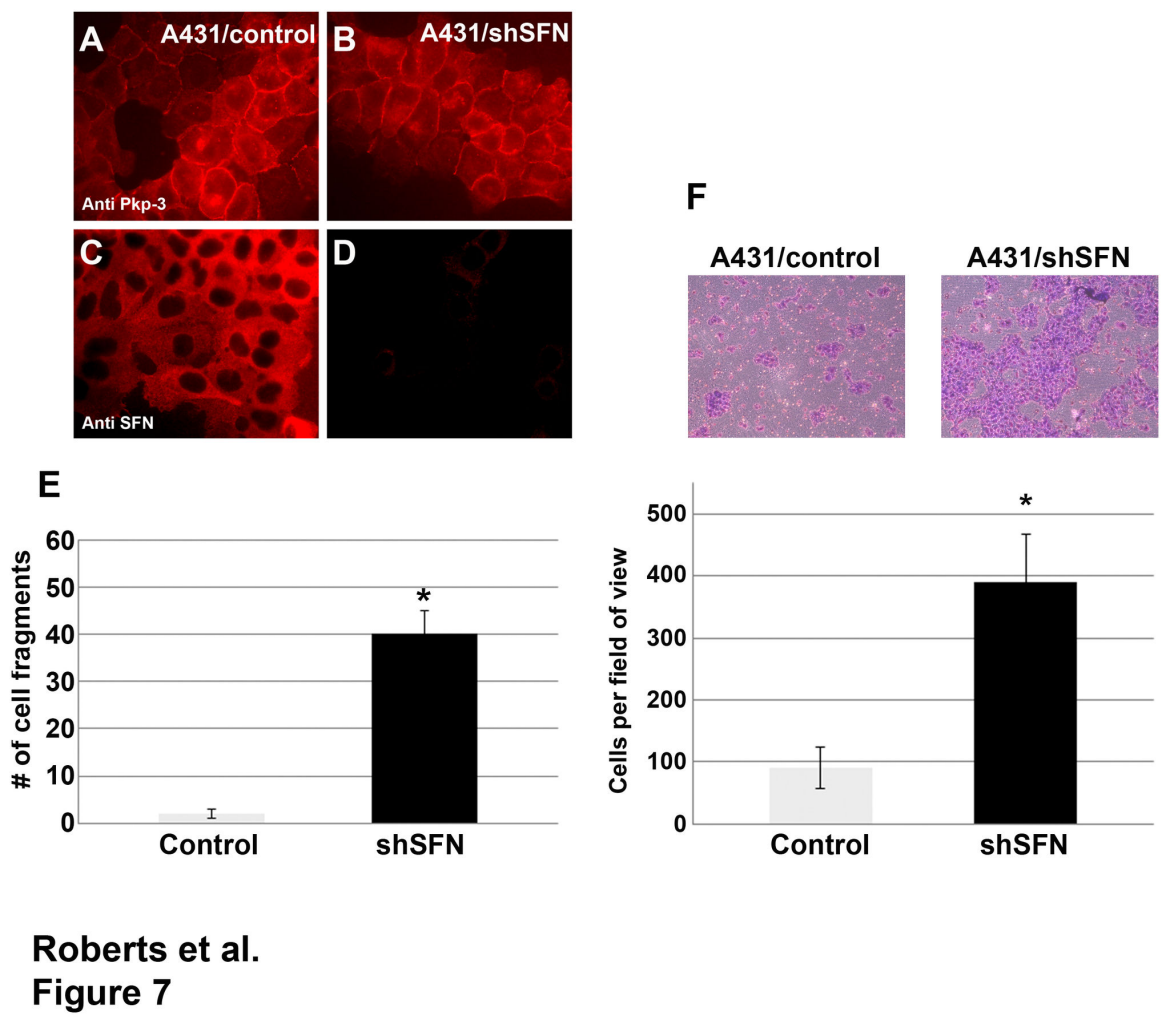
Decreased stratifin expression leads to decreased desmosomal adhesion and increased cell migration. A431 cells expressing a control shRNA (A431/control) (panels A and C) and A431 cells expressing shRNA targeting stratifin (panels B and D) were immunostained using anti plakophilin-3 (panels A and B) and anti stratifin antibodies (panels C and D). A431 cells stably expressing a control shRNA or shSFN targeting stratifin were subjected to a dispase adhesion assay (panel E). A431/control epithelial sheets remained intact following mechanical shearing while A431 cells expressing shSFN fragmented more readily after mechanical shearing. The graph represents triplicate cultures (p-value < 0.0001; Student’s t-test). Cell migration was measured using a Boyden chamber assay (panel F). Cells were plated on top of a filter insert and 24 hours later non migratory cells were removed while cells that migrated to the bottom of the filter were fixed, stained and counted. The assay was done in triplicate and the graph depicts the average number of cells per field of view (p-value < 0.0001, Student’s t-test).

While stratifin has been shown to interact with a large number of substrate proteins and could potentially affect diverse cell behaviors, we chose to test the ability of stratifin knock down to affect desmosomal cell-cell adhesion using the dispase adhesion assay. A431 cells expressing shRNA targeting stratifin and control shRNA were generated and desmosomal adhesion was assessed using the dispase assay (Figure 7E). Stratifin knock down did not affect the steady state localization of plakophilin-3 at cell-cell borders (Figure 7A-D). Control A431 cells maintained tight cell-cell adhesion and epithelial sheets were maintained after removal from the culture dish and light mechanical shearing. In contrast, decreased stratifin expression resulted in decreased cell-cell adhesion and an increase in fragmentation in the dispase adhesion assay indicating decreased desmosomal adhesion [32,34]. We expect that this is due to the increased exchange of endogenous plakophilin-3 with the desmosomal plaque in the absence of stratifin complexes in the cytosol.

Previous studies have demonstrated that decreased expression of plakophilin and disruption of desmosomal adhesion contributes to increased cell migration [28,35]. Since stratifin expression resulted in decreased desmosomal adhesion, we expected cells expressing shRNA targeting stratifin (shSFN) would also display increased migration. We compared the migratory capacity of A431 cells expressing shSFN to that of A431 cells expressing a control shRNA using a modified Boyden chamber assay [27,28]. Cells expressing shRNA targeting stratifin were significantly more migratory than control A431 cells consistent with the observation that desmosomal adhesion is decreased in these cells (Figure 7F).

## Discussion

Desmosomal adhesion plays an important role in the maintenance and homeostasis of epithelial tissues. These adhesive junctions are points of continuity for the keratin intermediate filament cytoskeleton and provide cells in an epithelium a mechanism to withstand mechanical stress. Decreased desmosomal adhesion is widely believed to result in epidermal blistering [36]. On the other hand, mechanisms must exist to modulate the strength of cell-cell adhesion in order to allow controlled migration of epithelial cells in a tissue. For example re-epithelialization during wound healing, keratinocytes change their adhesive contacts with neighboring keratinocytes to allow cells at the leading edge to migrate into the wound area. The pathways that coordinate desmosome dynamics are poorly understood. We reasoned that proteins that interact with desmosomal plaque proteins might play a role in regulating desmosome dynamics. Here we identified stratifin as a plakophilin-3 interacting partner.

Plakophilins play an important role in desmosome assembly and maintenance. In addition to their roles in desmosome assembly, plakophilin-1 and plakophilin-3 have been demonstrated to become incorporated into stress granules in response to different stimuli, and plakophilin-1 interacts with translation elongation factors to affect mRNA translation [37,38]. Here we have demonstrated that plakophilin-3 is associated with stratifin in the cytoplasm, and its association with stratifin affects plakophilin-3 exchange with the desmosomal plaque. We did not observe plakophilin-3 or stratifin localization at stress granules or other cytoplasmic structures in A431, HaCat or other oral HNSCC cell lines examined. However, the role of stratifin in regulating plakophilin-3 incorporation into stress granules remains to be examined. FRAP analysis of plakophilin-3/GFP localized to the cytosol revealed complete and rapid fluorescence recovery (data not shown) suggesting that the plakophilin-3 present in the cytosol is not anchored to subcellular structures.

It was previously shown that localization of desmosome components were altered in the hair follicles of mice heterozygous ER/+ mice compared to wild type mice [17]. These mice display reduced anchorage of the telogen hair and these authors suggest altered stratifin expression results defects in desmosomal adhesion and are at least partially responsible for the hair follicle defect. Interestingly, we did not observe any significant changes in desmosomal component localization in our cultured cells when stratifin expression was reduced using short hairpin RNAs targeting stratifin. It is possible that there may be subtle changes in desmosome ultrastructure in cells with reduced stratifin expression though these studies have not been completed.

In our initial proteomics screen for plakophilin-3 interacting partners we detected tryptic peptides that matched stratifin. However we failed to detect other 14-3-3 peptides even though other 14-3-3 proteins are ubiquitously expressed [39]. While this interaction was robustly detected in the cell lines examined here, this does not exclude the possibility that plakophilin-3 could interact with other 14-3-3 family members in additional cell types and these experiments are currently underway in our laboratory. It will be particularly interesting to determine if other plakophilin isoforms interact with various 14-3-3 family members and if these interactions regulate the subcellular localization of additional plakophilin isoforms. For example, plakophilin-1 is prominently localized to the nucleus in cultured cells however the nuclear localization determinants are unknown [24]. Here we have demonstrated that stratifin interacts with plakophilin-3 but not with plakophilin-1 or -2. It was previously shown that plakophilin-2 possesses a C-TAK1 phosphorylation consensus site. It was proposed that phosphorylation at serine 82 promoted the binding of 14-3-3 and resulted in the localization of plakophilin-2 to the nucleus in transiently transfected SCC-9 cells [40]. Together these findings suggest there is some specificity of plakophilin association with 14-3-3 family members and 14-3-3 proteins are likely to regulate plakophilin localization or function.

14-3-3 binding to many partners includes a phosphoserine within the binding site. Here we demonstrated that plakophilin-3 is phosphorylated at serine 285 using a phospho specific antibody. Mutation of plakophilin-3 serine 285 to alanine prevented the association of plakophilin-3 with stratifin. Unfortunately, mutation of serine 285 to aspartic acid (S285D), in an attempt to generate a phospho mimetic was unsuccessful. While others have used this approach for other phospho-proteins this mutation did not demonstrate increased stratifin interaction. Plakophilin-3 S285D expressed in A431 cells did not co-immunoprecipitate more stratifin. Although the kinase that is responsible for phosphorylation at serine 285 is unknown, the activity of the kinase involved is likely to play a role in stability of the desmosomal plaque by affecting the association of plakophilin-3 with stratifin. After analyzing the sequence surrounding serine 285, we suspected protein kinase C as the potential kinase. Recent reports have suggested that PKCα plays an important role in regulating desmosome assembly and maintenance [41,42]. However when PKC was activated using phorbol-12-myristate-13-acetate, we found no increase in the phosphorylation of plakophilin-3 at serine 285, no effect on the kinetics of the incorporation of plakophilin-3 into the desmosomal plaque and no increase in the interaction of stratifin with plakophilin-3. Identification of the relevant kinase is ongoing in our laboratory.

**Figure 8:**
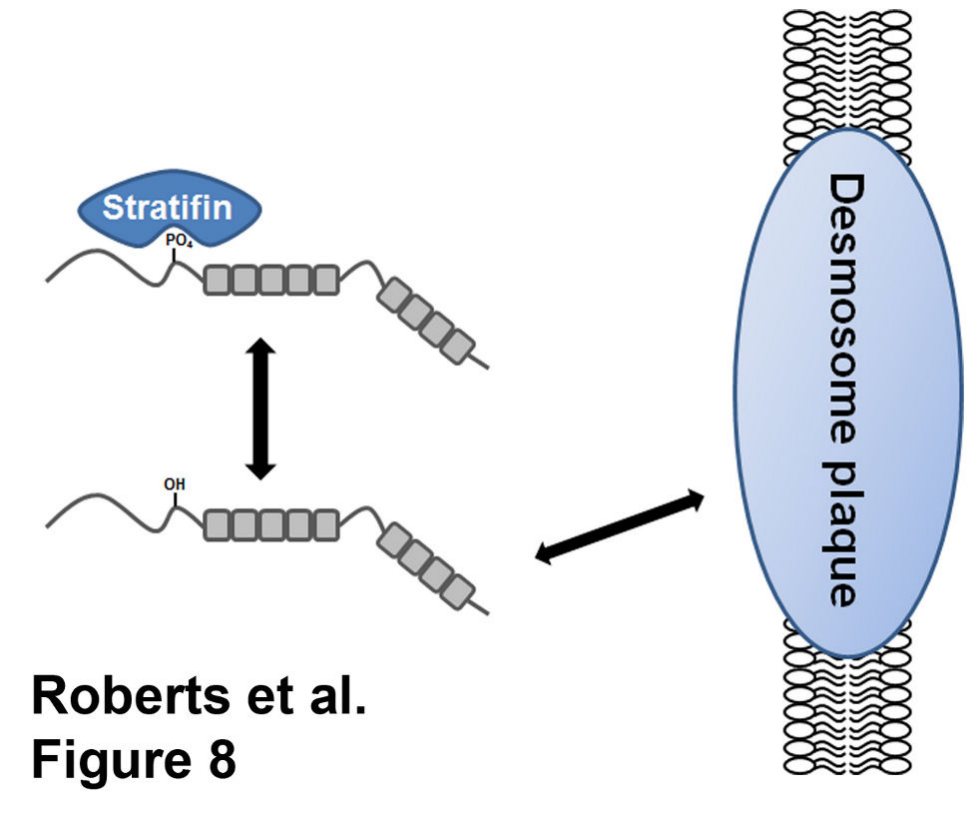
Stratifin association with plakophilin-3 limits the exchange of plakophilin-3 with the desmosomal plaque. The association of stratifin with plakophilin-3 occurs through phosphorylation of serine 285 and limits the ability of cytoplasmic plakophilin-3 to exchange with the plakophilin-3 present at the desmosome. Decreased plakophilin-3 exchange at the desmosome is correlated with increased desmosomal adhesion and decreased cell migration.

Identification of a plakophilin-3/stratifin complex in the cytoplasm and the increased exchange of plakophilin-3 S285A mutant with the desmosomal plaque leads us to propose a model in which regulation of the cytoplasmic pool of plakophilin-3 affects desmosomal adhesion. These interesting results and provide insights as to how epithelial cells maintain desmosomal adhesion during physiologic epithelial tissue remodeling. These mechanisms allow adjustments in desmosomal adhesive strength while still maintaining epithelial homeostasis to be maintained. Based on our findings we propose a model (Figure 8) in which stratifin association with plakophilin-3 in the cytosol sequesters this desmosomal plaque protein and restricts its exchange with the existing desmosomal plaque structures. Our model predicts that increasing the exchange of plakophilin with the desmosomal plaque can act to destabilize the adhesive strength of the junction and altered adhesive contacts with neighboring cells. Changes in adhesive character can promote changes in migratory behavior. Additionally, we predict phosphorylation of plakophilin-3 at serine 285 site acts to stabilize desmosomal adhesion by limiting the exchange of desmosomal plakophilin-3 with the cytoplasmic pool of plakophilin-3. Conversely, phosphatase activity may play an opposing role in this model and increased phosphatase activity would result in increased plakophilin-3 exchange with the desmosome. PP2A activity has been suggested to regulate 14-3-3 interactions with other binding proteins [43,44] and may be important in regulating plakophilin-3. Identification of the kinase and phosphatases acting upon plakophilin-3 and affecting desmosome dynamics will increase our understanding of the pathways regulating cell-cell adhesion.

## Supporting Information

Movie S1 **FRAP analysis of A431 cells expressing plakophilin-3/GFP and control shRNA.** A representative FRAP analysis of one cell-cell border showing plakophilin-3/GFP localization at the plasma membrane. Following photo ablation of the GFP signal, plakophilin-3/GFP recovery is followed until the fluorescence intensity reaches a plateau.
(WMV)

Movie S2 **FRAP analysis of A431 cells expressing plakophilin-3/GFP and shRNA targeting stratifin.** A representative FRAP analysis of one cell-cell border showing plakophilin-3/GFP localization at the plasma membrane. Following photo ablation of the GFP signal, plakophilin-3/GFP recovery is followed until the fluorescence intensity reaches a plateau.
(WMV)
